# Demystifying *Schrems II* for the cross-border transfer of clinical research data

**DOI:** 10.1093/jlb/lsab032

**Published:** 2021-10-23

**Authors:** Joseph Liss, David Peloquin, Mark Barnes, Barbara E Bierer

**Keywords:** GDPR, data privacy, data protections, clinical research, FISA, data transfer

## Abstract

The Courts of Justice of the European Union (CJEU) held in its July 2020 *Schrems II* decision that, in order for entities in other countries to import personal data from the European Economic Area (EEA), the importer must be able to provide data protections ‘essentially equivalent’ to those the EEA offers under its General Data Protection Regulation. The CJEU expressed particular concern that United States’ national security intelligence gathering laws prevent U.S.-based entities from providing such protections. This decision has sharply limited the sharing of clinical research data from the EEA to the United States. After describing the pertinent aspects of the *Schrems II* decision, this article evaluates U.S. national security intelligence gathering frameworks, including Section 702 of the Foreign Intelligence Surveillance Act and Executive Order 12333. The article then leverages recent draft guidance from the European Data Protection Board to explain how entities may be able to adopt widely used contractual and technical measures, such as data pseudonymization, to provide ‘essentially equivalent’ protections in the clinical research context.

## I. ARTICLE

Recent developments in European privacy law present significant challenges for European clinical researchers seeking to collaborate with researchers located in the United States and other countries located outside the European Economic Area (EEA). Under the General Data Protection Regulation (GDPR),^1^ EEA-based entities that seek to send personal data to the United States and other non-EEA jurisdictions must provide ‘essentially equivalent’ protections to those available in Europe.^2^ The Court of Justice of the European Union (CJEU) held in its July 2020 *Schrems II* decision that whether an entity importing personal data from the EEA (‘data importer’) can provide ‘essentially equivalent’ protections depends upon whether the laws of the importing country limit the ability adequately to protect personal data, including by affording sufficient rights to the persons to whom the personal data pertain (‘data subjects’).^3^ The CJEU expressed concern that the United States cannot provide such protections largely because of the U.S.’s national security intelligence gathering frameworks, including Section 702 of the Foreign Intelligence Surveillance Act (FISA) and Executive Order 12333 (E.O. 12333).^4^ In the wake of this decision, to send personal data to the United States, EEA-based data exporters must craft contractual agreements with U.S.-based data importers that ensure data subjects receive those ‘essentially equivalent’ protections and put in place other measures to ensure adequate protection.^5^ At the same time that the COVID-19 pandemic has underscored the need for international data sharing to support biomedical research, the *Schrems II* decision has complicated international, and particularly trans-Atlantic, data sharing, thereby frustrating progress of important biomedical research.

The biomedical research community has struggled to understand and apply the *Schrems II* decision, whose focus is on national security, an area that biomedical researchers and their institutions do not typically encounter. This article aims to demystify the *Schrems II* decision by laying out the requirements that *Schrems II* has established, evaluating U.S. intelligence laws and their potential effect on clinical research, and arguing that the combination of the European Union’s ‘standard contractual clauses’ (SCCs) and pseudonymization of research subject data will result in protections that are ‘essentially equivalent’ to those available under E.U. law. Other tools, including end-to-end encryption and Certificates of Confidentiality, may provide some additional benefit.

## II. DATA EXPORT REQUIREMENTS AFTER SCHREMS II

Both the Charter of Fundamental Rights of the European Union (‘the Charter’) and the GDPR govern the protection of Europeans’ personal data. The Charter provides the rights to respect for private and family life, the protection of personal data and the ability to obtain redress before an independent tribunal.^6^ E.U. courts read and interpret E.U. regulations ‘in the light of the Charter’.^7^ The GDPR extends expansively to all ‘personal data’ that EEA-based entities process or that foreign entities process in the course of offering goods and services to or monitoring the behavior of persons in the EEA.^8^

An EEA-based entity may not send personal data to a country located outside the EEA (referred to in the GDPR as a ‘third country’) unless the subject of the data receives protections essentially equivalent to those offered under E.U. law. While the European Commission may determine that a particular third country provides comparable protections to the GDPR,^9^ a process referred to as granting an ‘adequacy decision’, the United States and most other countries have not received such a decision. The E.U. had previously granted a partial adequacy decision for the United States under the GDPR’s predecessor in the form of the E.U.–U.S. Safe Harbor regime (the ‘Safe Harbor’), which permitted for-profit U.S. companies to self-certify compliance with certain data privacy standards, but the CJEU found the Safe Harbor invalid in 2015, in *Schrems I*.^10^ The U.S. subsequently received a partial adequacy decision applicable to for-profit companies that self-certified to the E.U.–U.S. Privacy Shield regime (the ‘Privacy Shield’).^11^ However, the CJEU invalidated that determination in *Schrems II*, finding that the Privacy Shield did not guarantee sufficient data protection.^12^ In particular, the CJEU found that U.S. intelligence gathering laws did not adhere to GDPR’s ‘principle of proportionality’ in failing to ‘lay down clear and precise rules governing the[ir] scope and application’ and provided insufficient ‘effective and enforceable rights and effective administrative and judicial redress’, especially for non-U.S. persons.^13^ In the aftermath of *Schrems II*, some commentators, such as Mr Schrems himself, have called for localizing data in the E.U., while others have critiqued localization as undermining both data privacy and economic development.^14^

For these reasons, data exporters seeking to export data from the EEA to the United States or to other countries lacking an adequacy decision must put in place another means to provide protections. One such mechanism, and that most commonly used, is the European Commission’s SCCs. The SCCs are European Commission-approved form contracts that permit data to flow between a data exporter located in the EEA and a data importer located outside of the EEA. The European Commission designed the clauses to afford adequate protection to personal data. E.U. authorities recently released proposed updates to the SCCs that are intended to rectify some long-recognized gaps in the coverage of the clauses.^15^ The draft SCCs were finalized in June 2021, after this article was accepted for publication; the finalized clauses are substantively similar to the draft.^16^

Historically, EEA-based data exporters have generally entered the SCCs with data importers without performing further analysis into the legal regime and data protection principles of the third country to which data are transferred, though the SCCs contain other important requirements.^17^ In the *Schrems II* decision, however, the CJEU made clear that data exporters cannot simply use the SCCs without additional diligence. Rather, the data exporter must evaluate ‘the relevant aspects of the legal system of that third country’, especially related to ‘any access by the public authorities of that third country to the personal data transferred’.^18^ The GDPR permits E.U. Member States to restrict GDPR rights when ‘such a restriction respects the essence of the fundamental rights and freedoms and is a necessary and proportionate measure in a democratic society to safeguard: national security.’^19^ In a November 2020 decision referred to as *La Quadrature du Net*, the CJEU interpreted a similar provision of a different E.U. law, holding that threats of terrorism could justify laws ordering electronic communications providers indiscriminately to retain certain information about, but not the contents of, large quantities of communications; however, retention of such collections must be time limited, and the collections subject to effective judicial oversight.^20^

The European Data Protection Board (EDPB), in draft guidance issued in November 2020 in response to the *Schrems II* decision,^21^ noted that data exporters must analyze whether public authorities in third countries access data only through programs which are ‘necessary and proportionate in a democratic society’.^22^ In performing this analysis, the EDPB wrote that data exporters should consider ‘objective factors’ rather than ‘subjective [factors] such as the likelihood of public authorities’ access to your data in a manner not in line with E.U. standards’.^23^ However, despite the statement that the likelihood of public authorities’ access should not be taken into account when conducting the analysis, the EDPB highlights that, when conducting the analysis, ‘objective factors’ that may be taken into account include precedents, legislation and practice ‘demonstrating that a third country authority will seek to access the data with or without the data importer’s knowledge … [or] will be able to access the data through the data importer or through direct interception of the communication channel.’^24^ Yet evaluating these factors calls for reliance on experience and history, rather than on the theoretical ability of a national security apparatus to obtain personal data. Furthermore, the implementing decision for the recently released draft SCCs similarly urges data exporters to ‘take into account the specific circumstances of the transfer’, including ‘practical experience indicating the existence or absence of prior instances of requests for disclosure from public authorities’ for that type of data or data recipient.^25^ The analysis cannot be static, but rather must be updated from time to time to evaluate changes in the laws of the importing country that may affect the protections afforded to personal data.^26^ Furthermore, the analysis must reach any contractor or sub-contractor that touches the data,^27^ such as email or cloud computing providers. The requirement is, therefore, essentially that the data exporter must evaluate an entire foreign legal system for these variables, and this has understandably proved a significant challenge to many EEA institutions, resulting in great confusion and the slowing—or suspending—of trans-national research collaborations.

In response to the *Schrems II* court’s finding that the U.S. legal system fails to afford equivalent protection to EEA residents, the U.S. government has argued that many E.U. Member States have even less intelligence oversight protections than does the United States. In particular, the U.S. government has highlighted how European Union Member States afford individuals more limited judicial review and provide less stringent regulation of both domestic and international clandestine intelligence collection than does the United States.^28^ Indeed, a 2015 report commissioned by the European Parliament found that unclear definitions, the lack of independent oversight and a lack of policies regarding when to inform targets about intelligence collection undercut protections in the Charter.^29^ However, in determining whether protections are ‘essentially equivalent’, the *Schrems II* court focused on a comparison to the rights enshrined in the GDPR and the Charter, rather than to the protections that E.U. Member States actually provide.^30^ Thus, data exporters must focus on the underlying rights—not the EEA’s current national security surveillance reality—in their analysis.

## III. U.S. INTELLIGENCE LAWS

Under the *Schrems II* framework, EEA-based exporters of personal data—including, in this context, clinical research data—must evaluate U.S. intelligence gathering laws and the risk of data capture by U.S. surveillance personnel, particularly under Section 702 of FISA and E.O. 12333. Many U.S. and European research institutions have struggled to understand these legal frameworks. We provide additional detail on that legal regime here, along with an explanation of why intelligence agencies are unlikely to target research data transferred to the United States.

Section 702 of the FISA was passed in 2008 and sought to authorize parts of President George W. Bush’s post-9/11 Terrorist Surveillance Program.^31^ It allows the National Security Agency (NSA) to collect intelligence on foreign targets located abroad from ‘electronic communication service provider[s]’ under a ‘certification’ that the Foreign Intelligence Surveillance Court (FISC) must approve annually.^32^ The certification must specify targeting, querying and data collection procedures that ensure only non-U.S. persons are targeted, minimize the capture of information about U.S. persons and ensure a ‘significant purpose of the acquisition is to obtain foreign intelligence information’.^33^ Once the FISC approves a ‘certification’, the NSA uses the approved methodology to generate lists of account identifiers, such as email addresses, and collects information flowing to or from one of those identifiers.^34^

FISA Section 702 provides limited protections for non-U.S. residents. The FISC’s annual certification focuses, as does the rest of Section 702, on whether U.S. intelligence agencies are targeting foreigners, as opposed to U.S. residents, not whether the foreigners targeted actually possess relevant intelligence information.^35^ Additionally, individuals targeted under Section 702 need not even be notified that the NSA is collecting data about them.^36^ The CJEU critiqued Section 702’s lack of both ‘limitations on the power it confers to implement surveillance programmes’ and ‘guarantees for non-US persons potentially targeted by those programmes’.^37^

Thus, researchers face two important questions before transferring clinical research data from the EEA to the United States: (i) from whom may the U.S. government collect data under FISA Section 702 and (ii) would the U.S. use FISA Section 702 to target clinical research data?

The definition of an ‘electronic communications services provider’ is potentially expansive. The definition primarily points to three other statutes, one of which is the definition of ‘electronic communication service’ under the Stored Communications Act (SCA).^38^ An electronic communication service, under the SCA, means ‘any service which provides to users thereof the ability to send or receive wire or electronic communications’.^39^ Courts have found the SCA definition covers a private employer that merely provides email services to its employees,^40^ which would cover the vast majority of entities conducting clinical research.

However, despite the potentially expansive reach of FISA Section 702, it seems likely—based on the limited public sources available—that the primary targets of NSA collection are major technology firms. For example, a 2014 report from the Privacy and Civil Liberties Oversight Board (PCLOB) implied that internet service providers were the key targets of much of the NSA’s data collection.^41^ The Edward Snowden leaks revealed that Microsoft, Google, Yahoo, AOL and Apple have provided the vast majority of the data that the NSA collected via its ‘downstream’ collections,^42^ formerly known as PRISM.^43^ The NSA supplements data collection from these entities with ‘upstream’ collections from ‘communications as they cross the backbone of the internet’.^44^ Thus, while firms operating their own email servers could become targets of Section 702 ‘downstream’ data collection under the statute’s expansive definitions, such targeting appears infrequent, at least based on information currently publicly available.

Moreover, the United Stated is unlikely to target clinical researchers for Section 702 collection. Section 702 authorizes collections when ‘a significant purpose … is to obtain foreign intelligence information’,^45^ a term that the statutory framework and court decisions suggest should be interpreted expansively.^46^ However, the NSA has, according to the FISC, indicated a narrower focus on queries ‘reasonably likely to retrieve foreign intelligence information’,^47^ a view reflected in the limitations Presidential Policy Directive 28 (PPD-28) placed on the gathering of signals intelligence (i.e., intelligence derived from electronic signals and systems, such as electronic communications).^48^ The 2019 National Intelligence Strategy indicated a similarly narrow focus on national security challenges.^49^ Clinical research studies are highly unlikely to involve data of these types, thus supporting a conclusion by exporters that an essentially equivalent level of protection can be available in the narrow ‘case’ of clinical research, without significant risk of disclosure for national security purposes.^50^

Additionally, Section 702 has at least some of the qualities that the CJEU has identified as essential to such national security legislation. For example, the FISC provides a level of independent oversight, the FISC’s certifications are time-limited and the threats targeted are national-security related, all factors the CJEU indicated were favorable in *La Quadrature du Net*.^51^ However, the CJEU found in *Schrems II* that ‘surveillance programmes based on Section 702 of the FISA and on E.O. 12333 are not covered by requirements ensuring, subject to the principle of proportionality, a level of protection essentially equivalent to that guaranteed’ by E.U. law.^52^ Thus, entities that the U.S. government successfully targets for data collection under Section 702 could not provide the adequate protections that *Schrems II* requires.

The other U.S. national security provision highlighted by the *Schrems II* decision, E.O. 12333, organizes the intelligence community and seeks to facilitate robust communication between intelligence agencies.^53^ The Order, which directs intelligence gathering that rests on the President’s inherent constitutional authority,^54^ seeks to strike ‘the proper balance between the acquisition of essential information and protection of individual interests’ and is permissive of more intrusive collection when directed against non-U.S. persons located abroad.^55^ The NSA Director serves as the U.S. intelligence community’s functional manager for collecting signals intelligence.^56^ Collecting data with the compelled assistance of private entities requires independent statutory authority and, thus, would not fall under E.O. 12333.^57^

Concerns about the volume and type of intelligence collection under E.O. 12333 led directly to President Obama’s Presidential Policy Directive 28 (PPD-28),^58^ which has remained ‘in full force and effect’ even after the end of the Obama presidency.^59^ PPD-28 imposed ‘appropriate safeguards for the personal information of all individuals, regardless of … nationality … or where that individual resides’. These safeguards include ‘minimize[ing] the dissemination and retention of personal information’, limiting data access to those with proper training and a need to know, and providing oversight.^60^ PPD-28 limits bulk signals intelligence (i.e., where the agency collects signals, such as phone or email communications, en masse, and then searches through the data for specific targets later^61^) to six specific threats: espionage, terrorism, the proliferation of weapons of mass destruction, cybersecurity threats, threats to the military and allies, and transnational criminal threats.^62^ The U.S. government has argued that PPD-28 is a substantial protection for the civil liberties of non-U.S. persons.^63^ However, the CJEU found PPD-28’s protections inadequate, since the NSA can collect bulk intelligence without specifying a particular target.^64^

Notably, however, as the U.S. government has argued, it is not clear that entities are putting their data at any greater risk of capture or review under programs authorized by E.O. 12333 merely by sending such data to the United States. First, no entity could be compelled to participate in bulk data collection under E.O. 12333, since such action requires separate statutory authority.^65^ While Section 702 provides such separate authority, as discussed above, the government is unlikely to use Section 702 to target clinical research data. Second, while the *Schrems II* court expressed particular concern about the NSA’s tapping of undersea cables that transfer communications to and from the United States,^66^ the Snowden leaks and other reporting have shown that the NSA collects data worldwide, such as when flowing between Europe, the Middle East, India and East Asia or when stored in China.^67^ A risk of data capture remains whenever and wherever data are sent electronically, thus challenging the CJEU’s concern that the act of transferring data to the United States increases the risk that such data will be accessed by U.S. authorities.^68^ Third, non-state actors, foreign intelligence services and other groups could theoretically seek to capture personal data at any point, suggesting that E.O. 12 333 does not pose a unique data capture risk.^69^

While not addressed by the CJEU in *Schrems II*, certain E.U. institutions have highlighted the CLOUD Act as an additional law that increases U.S. authorities’ access to data following transfer to the United States.^70^ The CLOUD Act permits the United States to access data that U.S.-based communications providers store *overseas* for purposes of criminal prosecutions. In particular, the CLOUD Act requires that, when federal, state or military prosecutors obtain a valid warrant, communications services providers must ‘disclose the contents of a wire or electronic communication … regardless of whether such communication … is located within or outside of the United States.’^71^ The presence of the CLOUD Act does not increase the risk that the U.S. government will access data transmitted to the United States because the CLOUD Act focuses on access to data held *overseas*. This means that the CLOUD Act would permit access to data stored abroad but have no effect on data stored in the United States, and thus, the CLOUD Act does not provide a rationale for limiting transfers of personal data to the United States.

## IV. STRATEGIES FACILITATING DATA TRANSFER

The EDPB guidance on data transfers post-*Schrems II* requires that entities exporting personal data from the EEA must put in place supplemental measures to safeguard the data. Fortunately, pseudonymization, a strategy the EDPB highlighted as an example of a supplemental measure, is commonplace in the clinical research environment.

The EDPB explains that *Schrems II* permits EEA-based data exporters to combine the SCCs with ‘supplementary measures’ that ensure an ‘essentially equivalent’ level of protection,^72^ while noting that the *Schrems II* decision ‘sets a high bar’.^73^ The supplementary measures must ‘address[] the specific deficiencies identified in [the data exporter’s] assessment of the legal situation in the third country’^74^ and ‘preclude potentially infringing access by preventing the authorities from identifying the data subjects, inferring information about them, singling them out in another context, or associating the transferred data with other datasets.’^75^ Data exporters must ensure that U.S. laws do not undermine their chosen safeguards.^76^ Notably, any contractors or vendors that touch the data likely must adopt the SCCs.^77^

Furthermore, the EDPB provides examples of potentially useful supplementary measures. For example, pseudonymization of data—where ‘the personal data can no longer be attributed to a specific data subject’—‘provides an effective supplementary measure’ if (i) the key to that data is ‘held exclusively by the data exporter and kept separately in a Member State’ or a third country with an adequacy decision and (ii) public authorities cannot use other information to re-identify the data.^78^ Pseudonymization is a technique routinely employed in clinical research to safeguard data, and typically, if data are collected by an investigator located in Europe and transferred in pseudonymized form to the United States, the key needed to link the pseudonymized data to the identity of the data subject will remain in the EEA. Data exporters may wish to consider additional security measures to protect the data key, such as limiting how often and with whom the key is shared and imposing appropriate contractual requirements on data recipients that they will not obtain a key and that if they do, they will immediately destroy it.

Of note for clinical researchers, the EDPB warned that ‘in many situations, factors specific to physical, physiological, genetic, mental, economic, cultural or social identity of a natural person … may allow the identification of that person’ even without ‘plain identifiers’.^79^ As the EDPB’s predecessor noted, genetic data inherently uniquely identifies an individual,^80^ making it more difficult to pseudonymize effectively. However, genotypic data can only be re-identified when linked to a reference database of genetic information containing the names of individuals. Therefore, imposing other technical and administrative safeguards can reduce, but not eliminate, the risk of re-identification of such data; given that it is impossible to entirely eliminate the risk of re-identification, it seems unlikely that transfers may only take place when no such risk exists. Additionally, there are ways to make genotypic data less helpful to, and thus less likely to be seized by, intelligence authorities; for example, removing identifying but non-research relevant phenotypic information may make genetic data more difficult to connect to a specific individual. This technique is also consistent with ‘the GDPR principle of “data minimisation”’.^81^

Examples of other supplementary measures include the following: First, end-to-end encryption can reduce the possibility that data will be re-identified, though the EDPB has expressed concern that the United States could use FISA Section 702 to obtain cryptographic keys.^82^ Second, since the NSA requests information flowing to or from particular ‘identifiers’, such as an email address,^83^ using separate mechanisms—such as separate servers or email addresses—to transmit personal data and research results would reduce the likelihood that the government will incidentally obtain personal data if it tries to obtain scientific information. Third, obtaining a Certificate of Confidentiality (CoC) may offer additional protection even though it may not prevent disclosure of data in response to a Section 702 request. CoCs prohibit disclosure of identifiable, sensitive information by researchers except in certain limited circumstances. One such circumstance permits certificate holders to release identifiable, sensitive information if federal, state or local law requires such release, unless the demand for release is part of a ‘proceeding’. As a result, a researcher who possesses a CoC and is faced with an applicable subpoena from a court for such identifiable, sensitive information is able to refuse complying with the subpoena because the court action is a ‘proceeding’.^84^ An NSA request under an FISC ‘certificate’ may not be considered a ‘proceeding’ unless the request recipient has challenged the request.^85^ Moreover, the NSA may request data from a vendor, such as an email provider, rather than the holder of the certificate, thus avoiding application of the CoC. In addition, the request recipient would have a complete release from liability for complying with the NSA’s request, reducing its incentive to challenge the NSA request.^86^ Nevertheless, the CoC could demonstrate that the data recipient has taken steps to shield the data from disclosure generally.

Pseudonymization, when combined with the SCCs and other supplementary measures, such as encryption and division of communication, will be helpful in common research scenarios. For example, registry studies may use a data coordinating center in the United States but have clinical sites collecting information across the world, including in the E.U., especially in the case of rare disease research. While the coordinating center often needs detailed medical information to facilitate research, patient identifying information—such as name, social insurance number, medical record number and other ‘direct identifiers’—is typically not required, thus making pseudonymisation a practical safeguard. Another common scenario is multi-regional drug trials that use a common laboratory in the United States to analyze all samples. While, as we note above, there are some questions as to whether it is possible to pseudonymize specimens that inherently contain genetic material, European data exporters can work with U.S.-based laboratories to impose contractual provisions and create protocols that prohibit U.S.-based researchers from using data to re-identify individuals.

If the data exporter cannot demonstrate the presence of adequate safeguards, an alternative mechanism that permits the cross-border transfer of data is obtaining the explicit consent of the data subject. First, the data exporter must inform the data subject about ‘the possible risks of such transfers for the data subject due to the absence of an adequacy decision and appropriate safeguards’.^87^ Second, explicit consent requires ‘an express statement of consent’, such as a written statement.^88^ However, ‘consent for data transfers [to countries without adequate levels of protection] that occur periodically or on an on-going basis is inappropriate’;^89^ thus, explicit consent could only be used for occasional or one-off transfers. Moreover, consent would not be an available basis for cross-border transfer in most secondary research for which data subjects are generally not asked to provide consent. The GDPR allows data transfers to third countries in other limited situations, such as when export is ‘necessary for the performance of a contract’ with or in the interest of the data subject or when ‘necessary for important reasons of public interest’.^90^ However, EDPB guidance construes such situations narrowly: for example, the public interest exception is available only when the public interest is shared by the third country and the EEA and transfers to perform a contract must be both ‘occasional’ and necessary.^91^ Even in the context of the COVID-19 pandemic, a clear example of a shared public interest between the EEA and third countries, the EDPB was careful to limit transfers of personal data for scientific research under the public interest derogation to ‘initial transfers’ following a ‘case-by-case’ analysis, suggesting that SCCs or another measure would be required for ongoing transfers for research purposes, such as a longitudinal study.^92^ Thus, using the SCCs to meet adequacy standards remains important in the clinical research context.

Finally, we note that other commentators are not as optimistic about cross-border data flows surviving *Schrems II*. Mr Schrems’ organization has said that SCCs are ‘dead’ as a mechanism to facilitate data flows to the United States, while Prof. Anupam Chander argues that small firms may well give up on U.S.–E.U. data transfers, as the SCCs require a miniature adequacy decision and consent comes with substantial restrictions.^93^ We take a more optimistic view for two reasons: First, the EDPB’s draft SCCs and guidance suggest that E.U. regulators are not seeking to stop all such data flows. Second, as the GDPR explicitly recognizes,^94^ E.U.–U.S. cooperation in scientific research offers great advantages to the E.U., and because of overarching, long-standing concerns for research ethics, researchers already make important efforts to protect the data and identity of research subjects. For these reasons, we see greater reason for optimism with respect to cross-border transfers for scientific research than with respect to transfers made for other purposes.

In summary, researchers can reasonably continue to transfer data or request that entities in the EEA transfer data, from the EEA to the United States following the *Schrems II* decision. While the NSA uses FISA Section 702 and E.O. 12333 to capture communications that those possessing foreign intelligence information send and receive, the NSA has no history of interest in research data. To ensure that data subjects receive ‘essentially equivalent’ protection in the United States as under the GDPR and the Charter, data exporters should, consistent with EDPB guidelines, ensure that data importers and their vendors agree to the SCCs and pseudonymize the research data, keeping the key in the EEA. By implementing additional protections and assessing that the likelihood is very low that U.S. intelligence agencies will actually target the relevant data, data exporters should be able to continue to transfer clinical research data to the United States without significant legal risk.

## Footnotes

1 Regulation (EU) 2016.679 of 27 Apr. 2016 [GDPR], 2016 O.J. [L 119] 1, https://eur-lex.europa.eu/legal-content/EN/TXT/PDF/?uri=CELEX:32016R0679.

2 C-311/18, Data Protection Comm’r v. Facebook Ire. Ltd. & Schrems [2020] [Schrems II], paras. 105, 162, at 31, 40, https://eur-lex.europa.eu/legal-content/EN/TXT/PDF/?uri=CELEX:62018CJ0311&from=EN.

3 Schrems II, para. 105, at 31.

4 Schrems II, paras. 168–202, at 41–46.

5 Schrems II, para. 105, at 31.

6 Charter of Fundamental Rights of the European Union (2000), Official Journal C364, 18 December, pp. 1–22 [Charter], arts. 7, 8, 47, 2000 O.J. (C 364) 10, 20 https://www.europarl.europa.eu/charter/pdf/text_en.pdf.

7 Schrems II, at 30 (July 16, 2020).

8 GDPR, art. 3, 2016 O.J. [L 119] at 32–33. The GDPR defines ‘personal data’ and ‘processing’ expansively. GDPR, art. 4, 2016 O.J. [L 119] at 33.

9 GDPR, Chapter V.

10 Schrems II, para. 42, at 18.

11 Schrems II, para. 43, at 18.

12 Schrems II, para. 199, at 45.

13 Schrems II, paras. 180, 188, 197, at 43–45.

14 See, e.g., Anupam Chander, *Is Data Localization a Solution for* Schrems II 1–2 (Georgetown University Law Center, July 27, 2020), https://scholarship.law.georgetown.edu/facpub/2300/ (‘Schrems himself suggested what seems to be an easy fix for companies transferring data outside Europe to the United States—do not. Leave the data in the EU…. Part II then asks whether such soft data localization advances either privacy or economic development, concluding that there are reasons to think that it undermines both.’).

15 European Commission Implementing Decision (EU) [Draft] of Nov. 12, 2020, https://ec.europa.eu/info/law/better-regulation/have-your-say/initiatives/12741-Commission-Implementing-Decision-on-standard-contractual-clauses-for-the-transfer-of-personal-data-to-third-countries.

16 European Commission Implementing Decision (EU) of June 4, 2021, https://eur-lex.europa.eu/eli/dec_impl/2021/914/oj?uri=CELEX:32021D0914&locale=en.

17 For example, data importers must provide information to data exporters that allow the data exporter to evaluate whether the data importer can comply with the SCCs; the SCCs provide for data subject rights as a third-party beneficiary to the contract; and data importers must inform data exporters if they are no longer able to comply with the SCCs, such as if law in the third country changes. See *Id*. at paras. (12–17), at 3–5.

18 Schrems II, paras. 105, 203(2) at 31, 46.

19 GDPR, art. 23, 2016 O.J. [L 119] at 26.

20 C-311/18, La Quadrature du Net et al. v. Premier ministre [2020] [La Quadrature du Net], para. 229(1), http://curia.europa.eu/juris/document/document.jsf?docid=232084&doclang=en.

21 The EDPB finalized its Guidelines after this article was accepted for publication in June 2021; the Guidelines are largely similar to the proposed version. *Recommendations 01/2020 on Measures That Supplement Transfer Tools to Ensure Compliance with the EU Level of Protection of Personal Data*, European Data Protection Bd. (June 18, 2021), https://edpb.europa.eu/our-work-tools/our-documents/recommendations/recommendations-012020-measures-supplement-transfer_en.

22 EDPB, SCC Recommendations, § 2.3(37), at 13.

23 EDPB, SCC Recommendations, § 2.3(42), at 14.

24 EDPB, SCC Recommendations, § 2.3(43), at 14. The EDPB’s footnote 45 directs readers to paragraph 43, in which the EDPB listed the factors we highlight here. EDPB, SCC Recommendations, § 2.3(42), at 14 & n.45. We thus conclude that a focus on the risk of data capture is an important consideration in this analysis.

25 European Commission Implementing Decision (EU) [Draft] of Nov. 12, 2020, *supra*, (20), at 5.

26 GDPR, art. 5(2), 2016 O.J. [L 119] at 36; EDPB, SCC Recommendations, § 2.6(62), at 18.

27 EDPB, SCC Recommendations, §§ 2.1(10), 2.3(31)–(33), at 9, 12.

28 Dept. of Comm., Information on U.S. Privacy Safeguards Relevant to SCCs and Other EU Legal Bases for EU-U.S. Data Transfers after Schrems II [White Paper], at 15–17 (Sept. 2020), https://www.commerce.gov/sites/default/files/2020-09/SCCsWhitePaperFORMATTEDFINAL508COMPLIANT.PDF.

29 European Union Agency for Fundamental Rights, Surveillance by Intelligence Services: Fundamental Rights Safeguards and Remedies in the EU 27, 58, 75 (Nov. 6, 2015), https://fra.europa.eu/en/publication/2015/surveillance-intelligence-services-volume-i-member-states-legal-frameworks.

30 See, e.g., Schrems II, at 46 (‘[T]he appropriate safeguards, enforceable rights and effective legal remedies required by [the GDPR] must ensure that data subjects … are afforded a level of protection essentially equivalent to that guaranteed within the European Union by [the GDPR], read in the light of the Charter of Fundamental Rights of the European Union.’).

31 Privacy and Civil Liberties Oversight Bd., Exec. Office of the President, Report on the Surveillance Program Operated Pursuant to Section 702 of the Foreign Intelligence Surveillance Act [PCLOB 702 Report] 17–20 (2014), https://documents.pclob.gov/prod/Documents/OversightReport/823399ae-92ea-447a-ab60-0da28b555437/702-Report-2.pdf.

32 50 U.S.C. § 1881a(a), (j)(3), (i)(1) (2018).

33 50 U.S.C. § 1881a(j)(3), (h)(2)(A) (2018).

34 PCLOB 702 Report, *supra*, at 32–33; White Paper, at 7, 14.

35 See 50 U.S.C. § 1881a(d)(1), (f)(1)(B); Prepared remarks by Rebecca J. Richards, director of the Civil Liberties and Privacy Office of the National Security Agency, before European Member States data protection authorities (Nov. 20, 2014), https://www.nsa.gov/Portals/70/documents/about/civil-liberties/resources/EU_DPA_Comissioners_Remarks_20141113.pdf.

36 See, e.g., Wikimedia Found. v. Nat’l Sec. Agency, 335 F. Supp. 3d 772 (D. Md. 2018) (precluding discovery of whether an organization was the target of surveillance under the state secrets doctrine).

37 Schrems II, para. 180, at 43.

38 50 U.S.C. § 1881(b)(4) (2018).

39 18 U.S.C. § 2510(12), (15) (2018).

40 See Fraser v. Nationwide Mut. Ins. Co., 352 F.3d 107, 115 (3d Cir. 2003); Shefts v. Petrakis, 758 F. Supp. 2d 620, 635 (C.D. Ill. 2010); see also Council on American-Islamic Rels. Action Network, Inc. v. Gaubatz, 793 F. Supp. 2d 311, 334 (D.D.C. 2011) (collecting cases).

41 PCLOB 702 Report, *supra*, at 33–34; see also Sneha Indrajuit et al., *FISA’s Section 702 & the Privacy Conundrum: Surveillance in the U.S and Globally*, Henry M. Jackson Sch. of Int’l Stud. (Oct. 25, 2017), https://jsis.washington.edu/news/controversy-comparisons-data-collection-fisas-section-702/ (‘Downstream collection focuses on Internet Service Providers ….’).

42 Barton Gellman & Laura Poitras, *U.S., British Intelligence Mining Data from Nine U.S. Internet Companies in Broad Secret Program*, Wash. Post (June 6, 2013), https://www.washingtonpost.com/investigations/us-intelligence-mining-data-from-nine-us-internet-companies-in-broad-secret-program/2013/06/06/3a0c0da8-cebf-11e2-8845-d970ccb04497_print.html; Steven Levy, *How the NSA Almost Killed the Internet*, Wired (Jan. 7, 2014), https://www.wired.com/2014/01/how-the-us-almost-killed-the-internet/; Julian Sanchez, *All About ‘About’ Collection*, Just Security (Aug. 28, 2017), https://www.justsecurity.org/40384/ado-about/.

43 *NSA Stops Certain Section 702 ‘Upstream’ Activities*, Release No: PA-014-18, Nat’l Security Agency (Apr. 28, 2017), https://www.nsa.gov/news-features/press-room/Article/1618699/nsa-stops-certain-section-702-upstream-activities/.

44 Office of Director of Nat’l Intelligence, Section 702 Overview (Accessed Oct. 19, 2020), https://www.dni.gov/files/icotr/Section702-Basics-Infographic.pdf.

45 50 U.S.C. § 1881a(h)(2)(A)(v) (2018).

46 50 U.S.C. §§ 1801(e), 1881(a) (2018); United States v. Turner, 2014 U.S. Dist. LEXIS 136375, *8 (N.D. Ill. July 25, 2014) (noting the need for deferential review).

47 FISC Opinion of Dec. 6, 2019, at 73–74 (FISA Ct. 2019), https://assets.documentcloud.org/documents/7202629/2019-702-Cert-FISC-Opinion-06Dec19-OCR.pdf (internal quotation marks omitted).

48 White House, Presidential Policy Directive—Signals Intelligence Activities, PPD-28 [PPD-28], at §§ 1(c), 2 (Jan. 17, 2014), https://obamawhitehouse.archives.gov/the-press-office/2014/01/17/presidential-policy-directive-signals-intelligence-activities. PPD-28 applies to Section 702. Office of the Director of Nat’l Intelligence, Status of Implementation of PPD-28: Response to the PCLOB’s Report [PPD-28 ODNI Report], at 5 (Oct. 2018), https://fas.org/irp/offdocs/pclob-ppd28-response.pdf.

49 See Office of Director of Nat’l Intelligence, National Intelligence Strategy of the United States of America 4–5 (2019), https://www.dni.gov/files/ODNI/documents/National_Intelligence_Strategy_2019.pdf;

50 See Schrems II, para. 134, at 35.

51 La Quadrature du Net, at para. 229(1) (permitting indiscriminate collection of traffic and location data ‘for the purposes of safeguarding national security,’ provided that the threat is ‘genuine and present or foreseeable’ and ‘such an instruction is [both] subject to effective review’ and ‘limited in time to what is strictly necessary’).

52 Schrems II, para. 178, at 42.

53 *Executive Order 12333: United States Intelligence Activities* [*E.O. 12,333*], Dept. of Justice (Aug. 10, 2013), https://it.ojp.gov/PrivacyLiberty/authorities/executive-orders; see Exec. Order No. 13,470, 73 Fed. Reg. 43,325 (Aug. 4, 2008); Exec. Order No. 13,355, 69 Fed. Reg. 53,593 (Sept. 1, 2004); Exec. Order No. 13,284, 68 Fed. Reg. 4,075 (Jan. 28, 2003).

54 U.S. Dept. of Just., Legal Authorities Supporting the Activities of the National Security Agency Described by the President 6–10 (Jan. 19, 2006); see also U.S. Const. art. II, §§ 1–3; Chicago & Southern Air Lines, Inc. v. Waterman S.S. Corp., 333 U.S. 103, 109, 111 (1948); In re Sealed Case No. 02–001, 310 F.3d 717, 742 (FISA Ct. of Rev. 2002).

55 *E.O. 12,333*, *supra*, at pts. 2.2, 2.4.

56 *E.O. 12,333*, *supra*, at pt. 1.3(b)(12)(A)(i).

57 White Paper, at 2, 16.

58 Daniel Severson, note, *American Surveillance of Non-U.S. Persons: Why New Privacy Protections Offer Only Cosmetic Change*, 56 Harv. Int’l L.J. 465, 466 (2015), https://harvardilj.org/wp-content/uploads/sites/15/562Severson.pdf.

59 Office of the Director of Nat’l Intelligence, Status of Implementation of PPD-28: Response to the PCLOB’s Report [PPD-28 ODNI Report], at 1 (Oct. 2018), https://fas.org/irp/offdocs/pclob-ppd28-response.pdf; see also PPD-28 ODNI Report, *supra*, at 4.

60 White House, Presidential Policy Directive—Signals Intelligence Activities, PPD-28 [PPD-28], at § 4 (Jan. 17, 2014), https://obamawhitehouse.archives.gov/the-press-office/2014/01/17/presidential-policy-directive-signals-intelligence-activities.

61 Nat’l Research Council, Bulk Collection of Signals Intelligence: Technical Options 33 (2015), https://www.nap.edu/read/19414/chapter/4#33.

62 PPD-28, *supra*, at § 2.

63 White Paper, at 19; PPD-28 ODNI Report, *supra*, at 2; Alexander W. Joel, *The Truth About Executive Order 12333*, Politico Mag. (Aug. 18, 2014), https://www.politico.com/magazine/story/2014/08/the-truth-about-executive-order-12333-110121.

64 Schrems II, para. 183, at 43.

65 White Paper, at 17.

66 Schrems II, para. 183, at 43 (‘That possibility, which allows, in the context of the surveillance programmes based on E.O. 12333, access to data in transit to the United States without that access being subject to any judicial review, does not, in any event, delimit in a sufficiently clear and precise manner the scope of such bulk collection of personal data.’).

67 See Philip Dorling, *Australian Spies in Global Deal to Tap Undersea Cables*, Sydney Morning Herald (Aug. 29, 2013), https://www.smh.com.au/technology/australian-spies-in-global-deal-to-tap-undersea-cables-20130828-2sr58.html; Barton Gellman & Ashkan Soltani, *NSA Infiltrates Links to Yahoo, Google Data Centers Worldwide, Snowden Documents Say*, Wash. Post (Oct. 30, 2013), https://www.washingtonpost.com/world/national-security/nsa-infiltrates-links-to-yahoo-google-data-centers-worldwide-snowden-documents-say/2013/10/30/e51d661e-4166-11e3-8b74-d89d714ca4dd_story.html; David E. Sanger & Nicole Perlroth, *N.S.A. Breached Chinese Servers Seen as Security Threat*, N.Y. Times (Mar. 22, 2014), https://www.nytimes.com/2014/03/23/world/asia/nsa-breached-chinese-servers-seen-as-spy-peril.html; see also Chander, *supra*, at 8 (‘As the Edward Snowden revelations helped demonstrate, foreign intelligence services today deploy malware, zero days, insider attacks and other exploits outside their own shores to target foreign intelligence targets.’).

68 The French data protection authority, CNIL, recently took the position in the context of reviewing a French government database that amassed pseudonymised health data for research purposes on a Microsoft Azure cloud platform that, even though Microsoft planned to store all data within the EEA, the *Schrems II* ruling meant Microsoft could not provide adequate protections under the GDPR; however, the French Administrative Supreme Court, the Conseil d’Etat, disagreed with this far-reaching position and noted that the data could continue to be stored on the Microsoft Azure platform, in large part due to the public interest in COVID-19 research and the lack of a sufficient technical alternative. Patrice Navarro & François Zannotti, *French Court refuses to suspend Microsoft’s hosting of a public health data lake despite CNIL opinion (the Health Data Hub case—Part 2)*, Lexology (Oct. 21, 2020), https://www.lexology.com/library/detail.aspx?g=6605e74e-25ad-44e7-abae-9d10ad667380; Catherine Stupp, *French Court Asks Microsoft for Safeguards Against U.S. Surveillance of Health Data*, Wall St. J. (Oct. 23, 2020 5:30 AM ET), https://www.wsj.com/articles/french-court-asks-microsoft-for-safeguards-against-u-s-surveillance-of-health-data-11603445400.

69 White Paper, at 3.

70 Letter from Andrea Jelinek, chair of the European Data Protection Board, to Members of the European Parliament in response to a letter regarding the agreement between the UK and the USA on Access to Electronic Data for the Purpose of Countering Serious Crime (June 15, 2020), https://edpb.europa.eu/sites/edpb/files/files/file1/edpb_letter_out_2020-0054-uk-usagreement.pdf.

71 18 U.S.C. §§ 2703(a), 2713 (2018). Prior to the CLOUD Act, it was not clear if the SCA applied beyond U.S. boarders. Microsoft v. United States, 829 F.3d 197 (2d Cir. 2016).

72 EDPB, SCC Recommendations, § 2.2(23), at 2, 11 (Nov. 10, 2020).

73 EDPB, SCC Recommendations, § 3(64), at 19.

74 EDPB, SCC Recommendations, Annex 2(70), at 21.

75 EDPB, SCC Recommendations, Annex 2(74), at 21–22.

76 EDPB, Essential Guarantees, § 4(53), at 15; EDPB, SCC Recommendations, § 2.3(34), at 13.

77 European Commission Implementing Decision (EU) [Draft] of 12 November 2020, at para. 1, https://ec.europa.eu/info/law/better-regulation/have-your-say/initiatives/12741-Commission-Implementing-Decision-on-standard-contractual-clauses-for-the-transfer-of-personal-data-to-third-countries; *id* at ANNEX, § 2, cl. 4(b); *id*. at ANNEX, § 2, Module One, cl. 1.7; EDPB, SCC Recommendations, § 2.1(10), at 9.

78 EDPB, SCC Recommendations, Annex 2(80), at 23.

79 EDPB, SCC Recommendations, Annex 2(81), at 23–24.

80 Article 29 Data Protection Working Party, Opinion 4/2007 on the Concept of Personal Data 8–9 (June 20, 2007), https://ec.europa.eu/justice/article-29/documentation/opinion-recommendation/files/2007/wp136_en.pdf.

81 EDPB, SCC Recommendations, § 2.1(11), at 9.

82 EDPB, SCC Recommendations, Annex 2, at 22.

83 See PCLOB 702 Report, *supra*, at 32–39 (describing the process of ‘tasking’ individual identifiers).

84 42 U.S.C. § 241(d)(1) (2018).

85 50 U.S.C. § 1881a(i)(4) (2018).

86 50 U.S.C. § 1881a(i)(3) (2018).

87 GDPR, art. 49(1)(a), 2016 O.J. [L 119] at 64.

88 European Data Protection Bd., Guidelines 05/2020 on Consent Under Regulation 2016/679 [EDPB, Consent Guidelines], § 4(91), (93), 1, 20–21 (Nov. 10, 2020), https://edpb.europa.eu/sites/edpb/files/files/file1/edpb_guidelines_202005_consent_en.pdf.

89 EDPB, Consent Guidelines, § 4(91), at 20 n.4.

90 GDPR, art. 49, 2016 O.J. [L 119] at 64.

91 European Data Protection Bd., Guidelines 02/2018 on Derogations of Article 49 Under Regulation 2016/679, §§ 2.2, 2.4, 8, 10–11 (May 25, 2018), https://edpb.europa.eu/our-work-tools/our-documents/directrices/guidelines-22018-derogations-article-49-under-regulation_en.

92 European Data Protection Bd., Guidelines 03/2020 on the Processing of Data Concerning Health for the Purpose of Scientific Research in the Context of the COVID-19 Outbreak, § 7(67), (68), 12 (Apr. 21, 2020), https://www.huntonprivacyblog.com/wp-content/uploads/sites/28/2020/04/edpb_guidelines_202003_healthdatascientificresearchcovid19_en.pdf.

93 Chander, *supra*, at 4, 6.

94 See, e.g., GDPR, (33), (50), 2016 O.J. [L 119] at 6, 9 (recognizing exceptions under the GDPR for research).

